# Different Dilutions of Mesobotox in Facial Rejuvenation: Which is Better?

**DOI:** 10.1007/s00266-024-04029-7

**Published:** 2024-05-07

**Authors:** Samar Abdallah Salem, Wael Mohamed Seoudy, Nesma Saber Abd El-Rahman, Ahmed Abdelfattah Afify

**Affiliations:** 1 Faculty of Medicine, Ain Shams University, Cairo, Egypt; 2 Faculty of Medicine, Misr University for Science and Technology, Giza, Egypt; 3 Misr University for Science and Technology, Giza, Egypt

**Keywords:** Dilutions, Mesobotox, Facial, Rejuvenation

## Abstract

**Background:**

Microbotulinum toxin A treatment is a technique of delivering multiple intradermal injections of diluted botulinum toxin type A into the dermis or the interface between the dermis and the superficial layer of the facial muscles to preserve the facial mobility. The current study aimed to evaluate and compare the clinical effect of different dilutions of microbotulinum toxin A in periorbital and mid-facial rejuvenation.

**Methods:**

This randomized prospective interventional study included 30 female patients with different types of wrinkles in periorbital and/or mid-face. Patients were divided into three groups: group I (10 patients): 100U botulinum toxin in 5 ml saline, group II (10 patients): 100U botulinum toxin in 7 ml saline and group III (10 patients): 100U botulinum toxin in 10 ml saline.

**Results:**

A statistically significant better global esthetic improvement scale (GAIS) scores after 1 month were observed in group I compared to groups II and III. Also, after 6 months better GAIS scores were observed in group I compared to group II and in group II compared to group III. Assessment of different esthetic parameters measured by the Antera 3D camera revealed a statistically significant improvement in all parameters (periorbital and mid-face) in group I and in most of parameters (periorbital and mid-face) in groups II and III with more evident improvement after 1 month compared to after 6 months.

**Conclusion:**

Intradermal microbotulinum toxin A is a cost-effective method for improving periorbital and mid-face wrinkles with a better effect of 1:5 than 1:7 and 1:10 dilutions.

- Facial wrinkles possess a great burden on patients' psychological status, and the emergence of novel rejuvenation technique with minimal side effects is necessary.
- MicroBoNT-A usage in the literature was through variety of dilutions and concentrations. Therefore, a conclusive and comparative study was essential to compare the effect of different microBoNT-A dilutions.
- In this context, the current study aimed to evaluate and compare the clinical effect of different dilutions of microBoNT-A in periorbital and mid-facial rejuvenation.

**Level of Evidence III:**

This journal requires that authors assign a level of evidence to each article. For a full description of these Evidence-Based Medicine ratings, please refer to the Table of Contents or the online Instructions to Authors www.springer.com/00266.

## Introduction

Facial aging is a gradual process which could be due to intrinsic and extrinsic causes. It results in the appearance of tissue ptosis, wrinkles, epidermal and dermal atrophy, dryness and enlarged pores [1].

The first signs of aging face are visible in third decade, when eyebrows start to descend as a result of increased skin laxity, gravitational force and repeated contractions of muscles in periorbital region [2]. Progression of aging process in the following decades produces additional changes in facial soft tissues and bony landmarks, leading to alterations in facial proportions [3].

The most prominent changes in middle third of the face refer to nose and cheeks. Thinning of nasal skin and subcutaneous tissue leads to increased prominence of bony and cartilaginous support structures. Additionally, nasal tip ptosis occurs. These changes in combination with recession of neighboring supporting structures lead to appearance of an increased nasal length [4].

Facial rejuvenation starts at a prophylactic strategy through preservation of skin health by regular sleeping, avoidance of stressful lifestyle and maintaining healthy diet. Natural dermatological emollients and topical applications help eliminate early aging signs. More advanced aging signs require more extensive medical treatment through injectable medications as botulinum toxin. Moreover, physical interventions as lasers and phototherapy prove helpful. Surgical interventions possess last line of treatment in resistant cases [5].

Microbotulinum toxin A (micro BoNT-A) treatment is a technique of delivering multiple microdroplet injections of diluted botulinum toxin type A into the dermis or the interface between the dermis and the superficial layer of facial muscles, glands and skin structures to preserve the facial mobility and natural beauty [6].

Facial wrinkles possess a great burden on patients' psychological status, and the emergence of novel rejuvenation techniques with minimal side effects is necessary. MicroBoNT-A usage in the literature was through variety of dilutions [7]. In this context, we aimed to evaluate and compare the clinical effect of different dilutions of microBoNT-A in periorbital and mid-facial rejuvenation.

## Materials and Methods

### Participants

This randomized prospective interventional study followed instructions of the Research Ethics Committee (FMASU MS 747/2020) and the Declaration of Helsinki.

Sample size: After reviewing literature, no previous similar study was done. So, being a pilot study, 10 cases per group were needed (total of 30 cases). Thirty female patients above 30 years with different types of wrinkles in periorbital and/or mid-face of class II or class III according to Glogau wrinkle scale were selected randomly in the period between March 2020 and November 2021 [8].

Patients with known hypersensitivity to botulinum toxin, patients with bleeding disorders or disorders of muscle activity (e.g., Myasthenia gravis), pregnancy or lactation, patients receiving aminoglycosides or calcium channel blockers, patients with previous cosmetic treatment for wrinkles within the past nine months and patients using topical antiaging preparation in the previous 3 months were excluded. Patients were divided into three equal groups, and each group was randomly assigned to a specific dilution of MicroBoNT-A. In group I (10 patients), we used 100U botulinum toxin diluted in 5 ml saline, in group II (10 patients), we used 100U botulinum toxin diluted in 7 ml saline, and in group III (10 patients), we used 100U botulinum toxin diluted in 10 ml saline.

## Clinical Evaluation

Each participant was subjected to:

- Signing an informed consent.
- Full history taking.
- Complete general examination.
- Local examination: wrinkle assessment using the Glogau classification [8].
- Assessment by digital photographs (Nikon D5200 camera, Japan) and Antera 3D camera (Miravex, Ireland): at day 0, 1 month and 6 months post-injection.

## Treatment Protocol

Topical anesthetic cream was applied 30 minutes before the procedure. Injection of microBoNT-A for facial wrinkles using botulinum toxin A (Neuronox, Korea) 100 units/vial was done. In the first group, 100 IU of botulinum toxin was diluted on 5 ml saline; thus, every 1 ml contained 20 IU of botulinum toxin. Then each 1 ml was divided among 2 insulin syringes (50 units type of insulin syringe). Every syringe contains 10 IU of botulinum toxin, and each small mark, in the syringe which is graduated into 50 small marks, contained 0.2 IU of botulinum toxin.

In the second group, 100 IU of botulinum toxin was diluted on 7 ml saline; thus, every 1 ml contained 14.28 IU of botulinum toxin. Then each 1 ml was divided among 2 insulin syringes. Every syringe contains 7.14 IU of botulinum toxin, and each small mark contained 0.14 IU of botulinum toxin.

In the third group, 100 IU of botulinum toxin was diluted on 10 ml saline; thus, every 1 ml contained 10 IU of botulinum toxin. Then each 1 ml was divided among 2 insulin syringes. Every syringe contains 5 IU of botulinum toxin, and each small mark contained 0.1 IU of botulinum toxin.

**Fig. 1:**
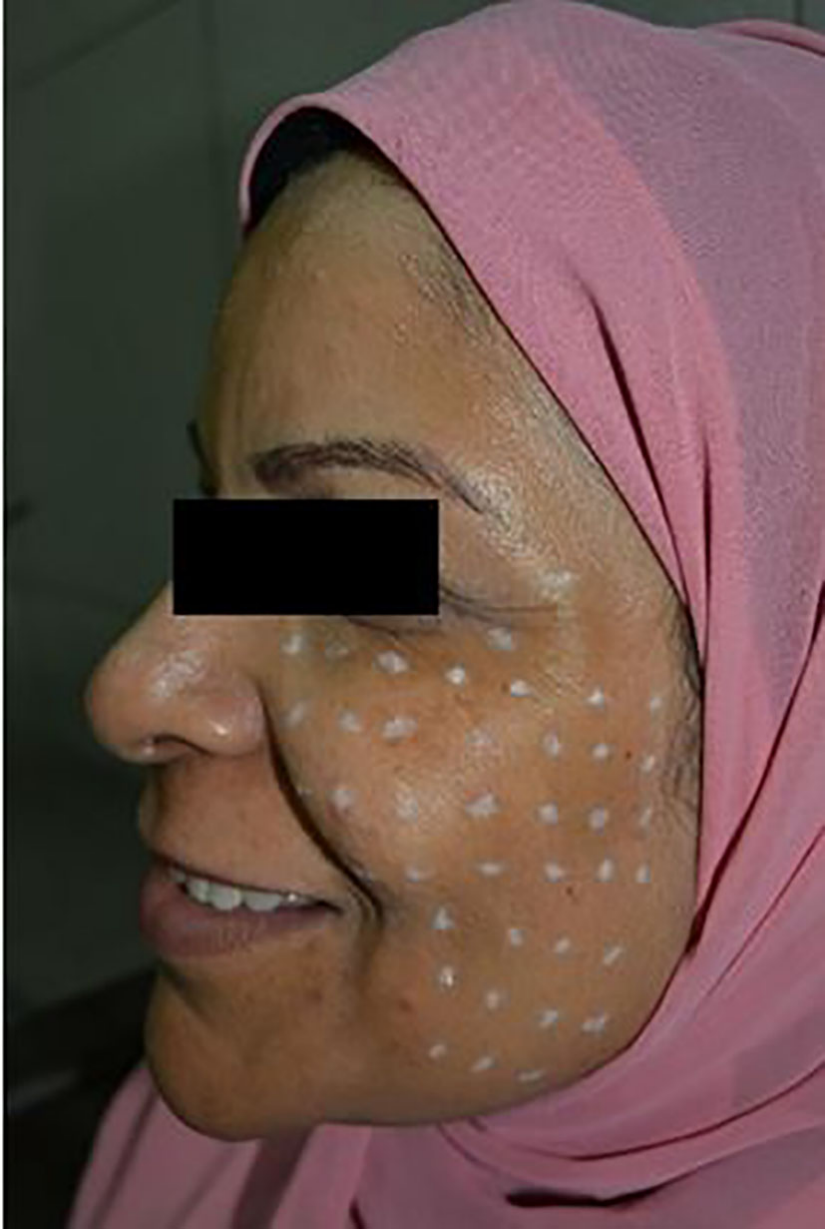
Areas for intradermal injection

Areas for injection were the periorbital, lateral cheek and anterior malar area (Figure 1). The injections were delivered intradermally using a 30G needle raising a tiny, blanched wheal at each point. Two marks from the syringe was injected per point with 1 cm distance between each point. Each point had 0.4 IU, 0.28 IU and 0.2 IU botulinum toxin for groups I, II and III. Each patient had about 30–33 injection points for each side of the face. No one needed touch-up, and only one injection session was done. Massage was avoided for the treated areas for 6 hours.

## Assessment and Evaluation

Patient satisfaction questionnaire was done according to 4 (0–3)-point Likert scale: 0 (unsatisfied), 1 (partially satisfied), 2 (moderately satisfied) and 3 (completely satisfied) [9].

Assessment through global esthetic improvement scale (GAIS), (1) Exceptional improvement: Excellent corrective result. (2) Very improved patient: Marked improvement of the appearance, but not completely optimal. (3) Improved patient: Improvement of the appearance, better compared with the initial condition, but a touch-up is advised. (4) Unaltered patient: The appearance substantially remains the same compared with the original condition. (5) Worsened patient: The appearance has worsened compared with the original condition [10]. Assessment of the response using Antera 3D camera and using the Glogau scale was done. Complications addressed with the patients during the treatment were recorded and analyzed.

## Statistical Methodology

Data were collected, revised, coded and entered to the Statistical Package for Social Science (IBM SPSS) version 20. The quantitative data with parametric distribution were presented as mean, standard deviations and ranges while those with nonparametric distribution were presented as median with inter-quartile range. Also, qualitative variables were presented as number and percentages.

Paired *T* Test was used to assess the statistical significance of the difference between two variable means in the same group. Chi-square test was used to examine the relationship between two qualitative variables. ANOVA test was used to compare means of two or more samples between more than two groups. *P*>0.05: nonsignificant and *P*≤ 0.05: significant.

## Results

The patient’s age in group I ranged from 43 to 55 years (Mean= 50 ± 4 years), in group II ranged from 45 to 57 years (Mean= 52 ± 4 years) and in group III ranged from 45 to 58 years (Mean= 53 ± 4 years). There was no statistically significant difference between the 3 groups as regards age (*P*= 0.337).

As regards patient’s satisfaction to treatment, in group I, after 1 month, 4 patients were moderately satisfied and 6 patients were completely satisfied while after 6 months, 2 patients were partially satisfied, 4 patients were moderately satisfied, and 4 patients were completely satisfied. In group II, after 1 month, 2 patients were partially satisfied, 7 patients were moderately satisfied and 1 patient was completely satisfied while after 6 months, 5 patients were partially satisfied, and 5 patients were moderately satisfied. In group III, after 1 month, 4 patients were partially satisfied and 6 patients were moderately satisfied while after 6 months, 4 patients were unsatisfied, and 6 patients were partially satisfied.

Comparison of patient satisfaction after 1 and 6 months in each group revealed significantly better satisfaction after 1 month than 6 months in group III only (*P*= 0.002) with no significant difference in the other two groups (*P*= 0.054 in group I, *P*= 0.117 in group II). Comparison between groups I and II revealed higher satisfaction after 1 month in group I (*P*= 0.041) and nonsignificant difference between the two groups after 6 months (*P*= 0.067). Comparison between groups I and III revealed higher satisfaction after 1 month (*P*= 0.006) and 6 months (*P*= 0.003) in group I. Comparison between groups II and III revealed statistically higher satisfaction after 6 months (*P*= 0.011) in group II and nonsignificant difference between the two groups after 1 month (*P*= 0.418).

A statistically significant better GAIS scores after 1 month were observed in group I compared to groups II and III. Also, after 6 months better GAIS scores were observed in group I compared to group II and in group II compared to group III (Table 1).

**Table 1:** GAIS scores in the three groups of the study after 1 and 6 months of treatment

Group I (1:5 dilution)	GAIS scores after 1 month				GAIS scores after 6 months				*P* value
	I	II	III	IV	I	II	III	IV	0.038
	–	5(50%)	5(50%)	–	–	–	7(70%)	3(30%)	
Group II (1:7 dilution)	GAIS scores after 1 month				GAIS scores after 6 months				
	I	II	III	IV	I	II	III	IV	0.038
	–	–	7(70%)	3(30%)	–	–	5(50%)	5(50%)	
Group III (1:10 dilution)	GAIS scores after 1 month				GAIS scores after 6 months				
	I	II	III	IV	I	II	III	IV	1.00
	–	–	1(10%)	9(90%)	–	–	1(10%)	9(90%)	
*P* value	0.023^ 0.038* 0.001** 0.107***				0.038^ 0.038* 0.107** 0.001***				

Using Chi-square test ^ across 3 groups

*GAIS* global esthetic improvement scale

*Between groups I and II

**between groups I and III

***between groups II and III

### Assessment of Treatment Response According to the Antera 3D Camera Measurements and Glogau Scale

**Fig. 2:**
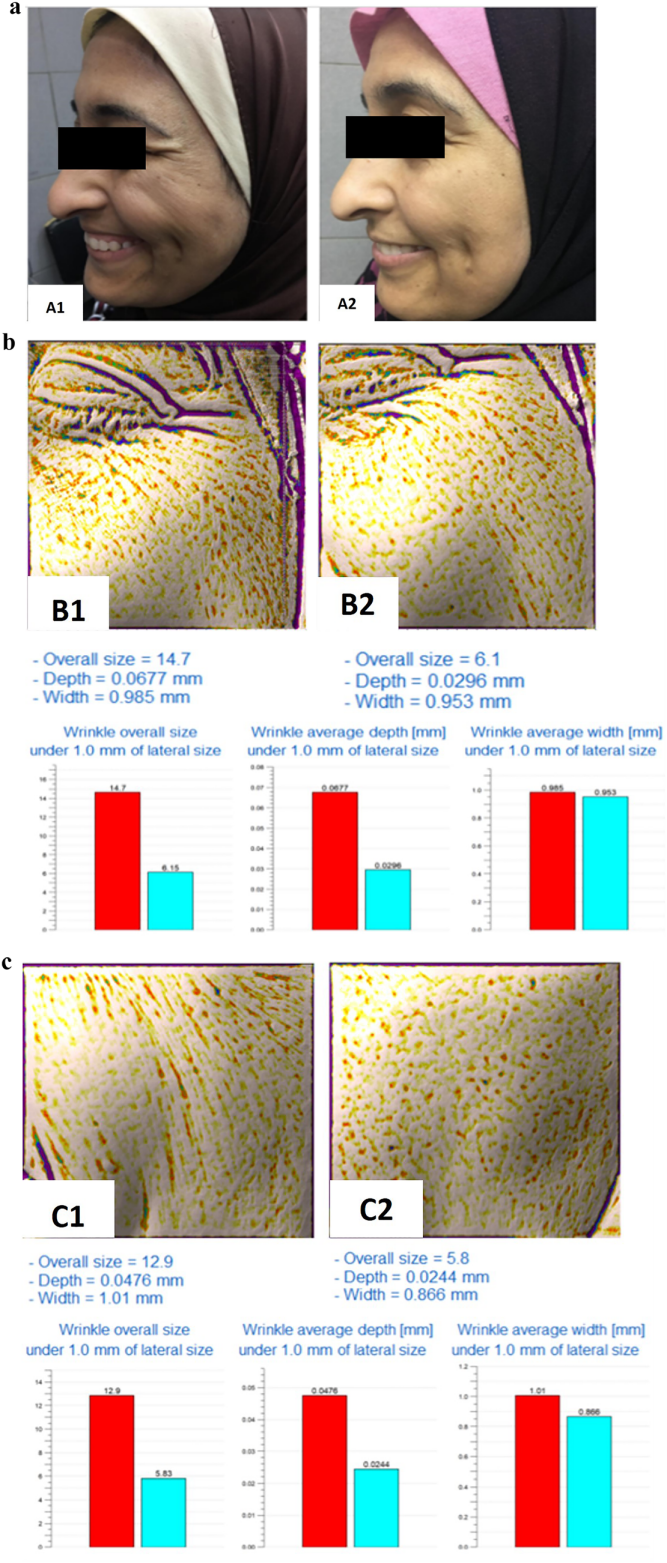
A 51-year-old female patient treated with microbotulinum toxin A (1:5 dilution). **a**1 Photograph before intervention. **a**2 Photograph 1 month after intervention showing very improved patient. **b**1 Antera camera report for periorbital wrinkles before intervention. **b**2 Antera camera report for periorbital wrinkles 1 month after intervention showing 70% improvement. **c**1 Antera camera report for mid-face wrinkles before intervention. **c**2 Antera camera report for mid-face wrinkles 1 month after intervention showing 71% improvement

Group I: Assessment of different esthetic parameters measured by the Antera 3D camera before and after 1 and 6 months of treatment revealed a statistically significant improvement in all the parameters (periorbital and mid-face) with more evident improvement after 1 month compared to after 6 months (Table 2). Periorbital and mid-face Glogau scale was 2 in 4 patients and 3 in 6 patients before treatment while after 1 month of treatment, 4 patients had a Glogau scale 1 and 6 patients had a Glogau scale 2, and this was statistically significant (*P*= 0.005) (Figure 2).

**Table 2:** Assessment of periorbital and mid-face response to microBoNT-A injection by Antera 3D camera measurements in group I

		Mean ± S.D.			*t*-value *P* value (Before and after 1 month)	*t*-value *P* value (Before and after 6 month)	*t*-value *P* value (After 1 month and after 6 month)
		Before treatment	After 1 month (% of improvement)	After 6 months (% of improvement)			
Wrinkle size (mm)	Periorbital	19 ± 5	5.7 ± 2.3	8.6 ± 2.2	11.598 **0.000**	6.195 **0.000**	3.868 **0.004**
		–	(70%)	(55%)	**0.000**	**0.000**	**0.031**
	Mid-face	20.7 ± 2.7	6 ± 2.3	7.6 ± 2.1	26.862 **0.000**	18.417 **0.000**	4.988 **0.001**
		–	(71%)	(64%)	**0.000**	**0.000**	**0.045**
Wrinkle depth (mm)	Periorbital	0.089 ± 0.021	0.034±0.014	0.057±0.015	16.195 **0.000**	7.079 **0.000**	6.633 **0.000**
		–	(62%)	(36%)	**0.000**	**0.000**	**0.021**
	Mid-face	0.098 ± 0.022	0.035±0.013	0.059±0.014	13.857 **0.000**	8.547 **0.000**	8.457 **0.000**
		–	(65%)	(41%)	**0.000**	**0.000**	**0.034**
Wrinkle width (mm)	Periorbital	0.86 ± 0.14	0.67 ± 0.098	0.72 ± 0.08	7.121 **0.000**	4.618 **0.001**	3.512 **0.007**
		–	(22%)	(16%)	**0.000**	**0.000**	**0.045**
	Mid-face	0.86 ± 0.14	0.62 ± 0.24	0.77 ± 0.09	6.223 **0.000**	4.003 **0.003**	2.285 **0.048**
		–	(28%)	(11%)	**0.000**	**0.000**	**0.024**
Indentation index	Periorbital	13.73 ± 2.88	9.51 ± 2.14	10.12 ± 2.1	4.913 **0.001**	5.054 **0.001**	3.301 **0.009**
		–	(31%)	(26%)	**0.000**	**0.000**	**0.046**
	Mid-face	16.39 ± 3.64	9.21 ± 2.05	10.06 ± 2.5	7.665 **0.000**	6.231 **0.000**	4.243 **0.002**
		–	(44%)	(39%)	**0.000**	**0.000**	**0.046**
Texture roughness	Periorbital	20.62 ± 7.13	11.06 ± 2.68	12.13 ± 2.64	5.848 **0.000**	5.523 **0.000**	2.705 **0.024**
		–	(46%)	(42%)	**0.000**	**0.000**	**0.048**
	Mid-face	19.62 ± 6.01	9.93 ± 3.15	10.74 ± 3.5	7.750 **0.000**	7.073 **0.000**	3.790 **0.004**
		–	(49%)	(45%)	**0.000**	**0.000**	**0.048**

Using paired *T* test

*SD* Standard deviation

*P* value ≤ 0.05 is significant

**Fig. 3:**
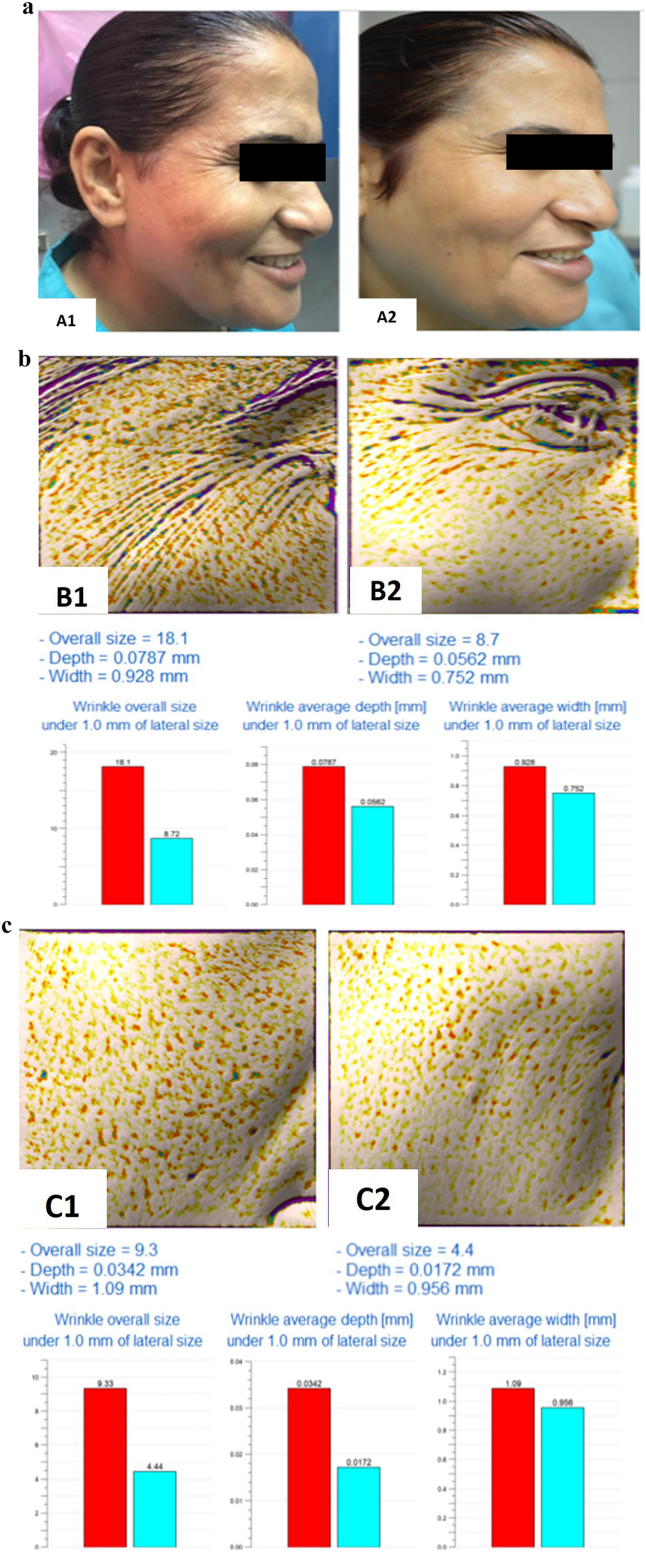
A 50-year-old female patient treated with microbotulinum toxin A (1:7 dilution). **a**1 Photograph before intervention. **a**2 Photograph 1 month after intervention showing very improved patient. **b**1 Antera camera report for periorbital wrinkles before intervention. **b**2 Antera camera report for periorbital wrinkles 1 month after intervention showing 42% improvement. **c**1 Antera camera report for mid-face wrinkles before intervention. **c**2 Antera camera report for mid-face wrinkles 1 month after intervention showing 41% improvement

Group II: Assessment of different esthetic parameters measured by the Antera 3D camera before and after 1 and 6 months of treatment revealed a statistically significant improvement in most of the parameters (periorbital and mid-face) with more evident improvement after 1 month compared to after 6 months. The only exception was the mid-face wrinkle depth and width which showed a statistically nonsignificant improvement before and after 1 and 6 months of treatment (Table 3). Periorbital and mid-face Glogau scale was 3 in 10 patients before treatment while after 1 month of treatment, 4 patients had a Glogau scale 2 and 6 patients had a Glogau scale 3, and this was statistically nonsignificant (*P*= 0.06) (Figure 3).

**Table 3:** Assessment of periorbital and mid-face response to microBoNT-A injection by Antera 3D camera measurements in group II

		Mean ± S.D.			*t*-value *P* value (Before and after 1 month)	*t*-value *P* value (Before and after 6 month)	*t*-value *P* value (After 1 month and after 6 month)
		Before treatment	After 1 month (% of improvement)	After 6 months (% of improvement)			
Wrinkle size (mm)	Periorbital	11.7 ± 7.1	6.8 ± 5.4	8.9 ± 6.7	5.196 **0.001**	3.667 **0.005**	3.765 **0.004**
		–	(42%)	(24%)	**0.000**	**0.000**	**0.015**
	Mid-face	18.9 ± 5.09	11.3±4.47	13.9±3.92	11.289 **0.000**	6.208 **0.000**	3.396 **0.008**
		–	(41%)	(27%)	**0.000**	**0.000**	**0.024**
Wrinkle depth (mm)	Periorbital	0.051 ± 0.032	0.041±0.034	0.046±0.038	4.436 **0.002**	3.697 **0.005**	2.934 **0.017**
		–	(20%)	(10%)	**0.000**	**0.000**	**0.021**
	Mid-face	0.075 ± 0.062	0.038±0.034	0.042±0.035	1.833 0.1	1.648 0.134	2.006 0.076
		–	(49%)	(44%)	**0.000**	**0.000**	**0.044**
Wrinkle width (mm)	Periorbital	0.95 ± 0.13	0.78 ± 0.17	0.82 ± 0.17	5.262 **0.001**	3.772 **0.004**	1.645 0.134
		–	(18%)	(14%)	**0.000**	**0.000**	**0.041**
	Mid-face	0.92 ± 0.14	0.79 ± 0.29	0.88 ± 0.39	2.008 0.076	0.463 0.655	2.089 0.066
		–	(15%)	(5%)	**0.000**	**0.000**	**0.016**
Indentation index	Periorbital	10.32 ± 2.14	8.09 ± 2.48	9.18 ± 2.43	4.184 **0.002**	3.397 **0.008**	4.260 **0.002**
		–	(22%)	(11%)	**0.000**	**0.000**	**0.022**
	Mid-face	11.81 ± 2.79	9.23 ± 2.22	10.32 ± 2.33	4.741 **0.001**	3.673 **0.005**	5.846 **0.000**
		–	(22%)	(13%)	**0.000**	**0.000**	**0.023**
Texture roughness	Periorbital	9.98 ± 2.69	7.54 ± 2.53	8.70 ± 2.49	3.869 **0.004**	2.603 **0.029**	4.620 **0.001**
		–	(25%)	(13%)	**0.000**	**0.000**	**0.019**
	Mid-face	12.01 ± 3.41	9.49 ± 2.32	10.58 ± 2.79	4.558 **0.001**	4.335 **0.002**	2.525 **0.033**
		–	(21%)	(12%)	**0.000**	**0.000**	**0.024**

Using paired *T* test

*SD* Standard deviation

*P* value ≤ 0.05 is significant

**Fig. 4:**
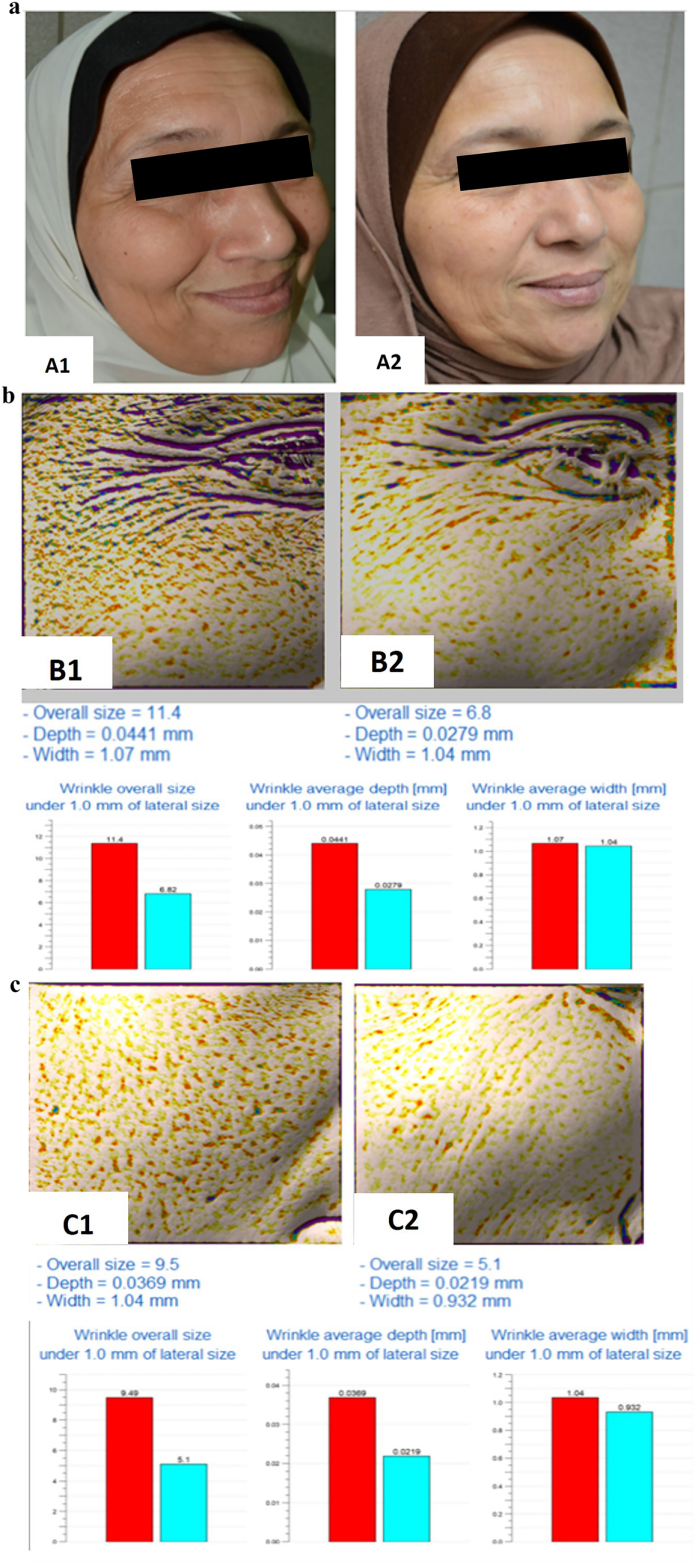
A 55-year-old female patient treated with microbotulinum toxin A (1:10 dilution). **a**1 Photograph before intervention. **a**2 Photograph 1 month after intervention showing improved patient. **b**1 Antera camera report for periorbital wrinkles before intervention. **b**2 Antera camera report for periorbital wrinkles 1 month after intervention showing 20% improvement. **c**1 Antera camera report for mid-face wrinkles before intervention. **c**2 Antera camera report for mid-face wrinkles 1 month after intervention showing 13% improvement

Group III: Assessment of different esthetic parameters measured by the Antera 3D camera before and after 1 and 6 months of treatment revealed a statistically significant improvement in most of the parameters (periorbital and mid-face) with more evident improvement after 1 month compared to after 6 months. The exceptions were in periorbital wrinkle depth that showed nonsignificant worsening of depth after 6 months and wrinkle width that showed a statistically nonsignificant improvement after 6 months of treatment. Moreover, mid-face wrinkle depth showed statistically nonsignificant improvement after 1 month of treatment and even worsening of depth after 6 months of treatment (Table 4). Periorbital and mid-face Glogau scale was 2 in 2 patients and 3 in 8 patients before treatment while after 1 month of treatment, 3 patients had a Glogau scale 2 and 7 patients had a Glogau scale 3, and this was statistically nonsignificant (*P*= 0.07) (Figure 4).

**Table 4:** Assessment of periorbital and mid-face response to microBoNT-A injection by Antera 3D camera measurements in group III

		Mean ± S.D.			*t*-value *P* value (Before and after 1 month)	*t*-value *P* value (Before and after 6 month)	*t*-value *P* value (After 1 month and after 6 month)
		Before treatment	After 1 month (% of improvement)	After 6 months (% of improvement)			
Wrinkle size (mm)	Periorbital	17.8 ± 7.5	14.3 ± 6.3	14.7 ± 7.5	5.649 **0.000**	2.741 **0.023**	0.387 0.707
		–	(20%)	(18%)	**0.000**	**0.000**	0.055
	Mid-face	20.8 ± 2.9	18.3±2.6	19.2±2.7	5.011 **0.001**	3.474 **0.007**	3.411 **0.008**
		–	(13%)	(8%)	**0.000**	**0.000**	**0.000**
Wrinkle depth (mm)	Periorbital	0.091 ± 0.022	0.049±0.022	0.15 ± 0.28	4.709 **0.001**	0.692 0.506	1.202 0.260
		–	(47%)	(-60%)	**0.000**	0.078	**0.000**
	Mid-face	0.15 ± 0.20	0.069±0.074	0.17±0.24	1.190 0.265	0.146 0.887	1.220 0.254
		–	(54%)	(-12%)	**0.000**	**0.000**	**0.000**
Wrinkle width (mm)	Periorbital	0.84 ± 0.29	0.68 ± 0.24	0.80 ± 0.12	5.463 **0.000**	0.528 0.610	2.212 0.054
		–	(20%)	(5%)	**0.000**	**0.01**	**0.021**
	Mid-face	0.79 ± 0.29	0.63 ± 0.25	0.72 ± 0.27	5.199 **0.001**	3.411 **0.008**	3.509 **0.007**
		–	(21%)	(9%)	**0.000**	**0.000**	**0.021**
Indentation index	Periorbital	13.01 ± 2.67	11.55 ± 2.36	12.14 ± 2.45	5.119 **0.001**	4.656 **0.001**	3.643 **0.005**
		–	(12%)	(7%)	**0.000**	**0.005**	**0.031**
	Mid-face	16.40 ± 2.79	14.33 ± 2.66	15.34 ± 2.91	13.796 **0.000**	6.857 **0.000**	8.773 **0.000**
		–	(13%)	(7%)	**0.000**	**0.000**	**0.030**
Texture roughness	Periorbital	13.19 ± 2.9	11.80 ± 2.68	12.51 ± 2.62	6.508 **0.000**	6.074 **0.000**	3.715 **0.005**
		–	(11%)	(5%)	**0.000**	**0.000**	**0.019**
	Mid-face	16.23 ± 2.76	14.05 ± 2.67	14.93 ± 2.96	8.353 **0.000**	5.651 **0.000**	4.898 **0.001**
		–	(14%)	(9%)	**0.000**	**0.000**	**0.034**

Using paired *T* test

*SD* Standard deviation

*P* value ≤ 0.05 is significant

Although it was not assessed either statistically or subjectively, there was a general observation of facial lifting in older rather than younger age. The latter observed less skin oiliness after than before treatment.

Frequency of side effects in included patients were minimal as only 1 patient complained of pain in group I, 1 patient complained of pain and another patient complained of erythema in group II while 2 patients complained of pain, 1 of erythema and 1 of edema in group III. Pain was encountered at time of injection while erythema and edema resolved few hours after injection.

## Discussion

Facial aging has a profound effect on individual's psychological state [11]. The main disadvantage of routine botulinum toxin injection is that the intramuscular injection causes complete paralysis of muscles of facial expression leading to a mask face appearance. For this reason, other techniques for use of botulinum toxin for esthetic purposes such as microBoNT-A should be explored for better esthetic outcomes [12].

MicroBoNT-A is the injection not of specific muscles, but the treatment of large areas of dermis with dilute botulinum toxin; however, no standardized method or dilution is agreed upon for the use of microBoNT-A [12].

Botulinum toxin has been shown to decrease sebum production, blocks production of eccrine sweat glands and improves fine lines and wrinkles by diminishing the pull of the facial depressors, resulting in an improvement of the facial contour [13–15].

In most of studies available in the literature evaluating any procedures for esthetic reasons, assessment of treatment response depends on either subjective methods such as grades or scales or objective methods such as biopsy taking. However, biopsy taking from a patient coming to perform an esthetic procedure carries a risk of scarring. To resolve this issue, we choose to assess the response to treatment by using the Antera 3D camera in combination with other methods such as patient satisfaction questionnaire and global esthetic improvement scale.

Antera 3D camera had several advantages. It helped us to accurately measure wrinkle size, texture, redness and pigmentation. Full analysis and report generation is done in less than 90 seconds. Measurements are highly accurate, with a ± 5% error. So, it is a good objective assessment tool. For this reason, and as the maximum effect reported for botulinum toxin injection is 6 months [16], we chose to do the second follow-up for the patients 6 months after the session to detect any subtle improvement induced by microBoNT-A, even if not visible by naked eye.

Patients’ satisfaction was higher after 1 month compared to after 6 months of treatment and was better in 1:5 dilution followed by 1:7 dilution and then 1:10 dilution. Similarly, treatment outcome assessment using GAIS score system revealed significantly better GAIS scores in 1:5 dilution compared to other two groups.

Although the Antera camera showed a significant improvement in the three studied groups, the 1:5 dilution showed a higher percentage of improvement in all parameters than the other two groups. In the 3 groups, the improvement was more evident after 1 month than after 6 months and this is similar to what happens with the regular intramuscular botulinum toxin A injection [7]. These findings suggest that very high dilutions of botulinum toxin (10 ml saline) may not give a satisfactory outcome possibly because of insufficient toxin concentration reaching the upper facial muscle fibers needed for their relaxation. It may even be followed by worsening of the wrinkles after complete resolution of the effect of microBoNT-A, which could be a normal consequence of aging effect on wrinkles after 6 months as the treatment was not satisfactory from the start.

De Oliveira et al. (2016) previously found a 66% rate of improvement of dynamic wrinkles in the lower portion of the orbicularis oculi muscle with one session of microdoses of botulinum toxin (1:5 dilution) [17].

Our finding of a significant improvement of skin texture roughness in the 3 studied dilutions, although better in the 1:5 than 1:7 and 1:10 dilutions, is in accordance with Cavallini and colleagues (2019) and Bertossi et al. (2019) study that showed a smoother, brighter and more elastic skin after microBoNT-A 1:5 dilution injected in facial wrinkles. However, their microBoNT-A was used in conjunction with fraxel laser and very low G prime HA. Thus, their results in this regard cannot be solely attributed to microBoNT-A alone. The improvement in texture present in our work could be attributed to tighter skin [18, 19].

In our study, the improvement in skin texture and tightness could also be attributed to increased collagen due to multiple needle puncture. This needs further assessment. Intradermal BoNT-A seems to be more suitable for patients with flat and flaccid muscles, which are often associated with loose skin. It can soften wrinkles; however, it was unable to improve glabellar lines, as the muscles in this area are strong and might respond better to intramuscular injection.

Petchngaovilai (2009) injected 261 patients with intradermal BoNT for mid-face lifting. A greater improvement of 24.9% of cases was achieved in cheek lifting while 65.52% showed moderate improvement and 9.58% reported minimal improvement [14].

In this study, a whole vial of BoNT-A was diluted with each specific hyperdilution. We found that this method is better in delivering a consistent result than taking a part of traditionally diluted BoNT and diluting it to the required concentration in the syringe. This latter method can result in non-homogenous muscle relaxation if BoNT is not well diluted with different concentration at each point of injection.

The side effects encountered were minimal and reversible, namely pain, erythema and edema, in very few patients at sites of injection. These side effects are in agreement with De Oliveira et al. (2016) who reported that 14% of cases had local pain while 4% had ecchymosis after microBoNT-A treatment [17].

Although delivering microdroplets superficially usually avoids complications, inadvertent diffusion to deeper muscle fibers, due to subdermal injection or delivery of larger volume, might result in total or partial paralysis. However, due to the lower neurotoxin concentration, these complications usually subside within 2–3 weeks: stiff immovable brow—due to diffusion into deep fibers of frontalis; weakness in neck movement—due to diffusion into sternocleidomastoid muscle; asymmetric smile and lower face atrophy—due to diffusion into depressor anguli oris, risorius or depressor labii inferioris; and festooning or “inanimate lower eyelid”—due to diffusion into orbicularis oculi particularly in patients with preexisting skin laxity. Few patients may also find the treatment uncomfortable due to multiple injection points [20, 21]. These side effects, though, were not encountered in the present work. In addition, we found that microdroplet injections can smoothen and tighten the skin and reduce both dynamic and static wrinkles possibly by inducing atrophy of sweat and sebaceous glands, weakening the superficial muscle fibers, thereby reducing the pulling and tethering force of facial muscles that form fine lines and wrinkles.

## Limitations

Limitations in this study include the small number of patients, inclusion of only females and not including patients from different ethnic groups.

## Conclusion

Intradermal microBoNT-A is a cost-effective method for improving periorbital and mid-face wrinkles with its different dilutions with a better effect of 1:5 than 1:7 and 1:10 dilutions.
